# Ceftriaxone modulates the acute corticosterone effects in local field potentials in the primary somatosensory cortex of anesthetized mice

**DOI:** 10.1038/s41598-019-56827-8

**Published:** 2019-12-30

**Authors:** Miguel Pais-Vieira, Carolina Kunicki, André Peres, Nuno Sousa

**Affiliations:** 1 Center for Interdisciplinary Research in Health, Institute of Health Sciences, Universidade Católica Portuguesa, Porto, Portugal; 2 Life and Health Sciences Research Institute (ICVS), School of Medicine, University of Minho, Braga, 4710-057 Portugal; 3 ICVS/3B’s, PT Government Associate Laboratory, Braga/Guimarães, 4710-057 Portugal; 4 Graduate Program in Neuroengineering, Edmond and Lily Safra International Institute of Neuroscience, Santos Dumont Institute, Macaiba, Brazil; 5 Clinical Academic Center (2CA-Braga), Braga, Portugal

**Keywords:** Sensorimotor processing, Stress and resilience

## Abstract

Stress responses are associated with elevations in corticosterone levels and, as a consequence, increases in glutamate in the central nervous system which can lead to neurological impairment. Ceftriaxone promotes glutamate transport and has been used to reduce glutamate toxicity, but so far it is not known whether ceftriaxone is able to reverse the effects of corticosterone administration. Here we describe the separate and combined effects of acute ceftriaxone and acute corticosterone administration in local field potentials (LFPs) recorded from the somatosensory cortex (S1) of anesthetized mice. For this, LFPs were recorded from groups of anesthetized mice injected with saline, corticosterone, ceftriaxone, or both. Comparison of global state maps, and their displacements, as measured by ratios of different frequency bands (Ratio 1: 0.5–20 Hz/0.5–45 Hz; and Ratio 2: 0.5–4.5 Hz/0.5–9 Hz) revealed distinct and opposite effects for corticosterone and for ceftriaxone. Corticosterone specifically increased the displacement in Ratio 2, while ceftriaxone decreased it; in addition, when both corticosterone and ceftriaxone were injected, Ratio 2 displacement values were again similar to those of the control group. The present results suggest that ceftriaxone and corticosterone modulate specific frequency bands in opposite directions and reveal a potential role for ceftriaxone in counteracting the effects of corticosterone.

## Introduction

Corticosterone is involved in stress responses and can induce multiple structural and physiological changes in the nervous system^1,2^. In some situations, these are part of adaptive stress responses, such as the *fight or flight response*^3^, while in other cases they are maladaptive and lead to impairment^1,2,4–7^. In rodents, corticosterone has been associated with changes in function and structure of different brain regions^8–11^. Stress, and more specifically corticosterone, also affects the primary somatosensory cortex (S1)^11,12^. However, our current knowledge about the effects of corticosterone in the primary somatosensory cortex is mostly the result of long term studies in the context of early life stressors and their effects in tactile processing in adult mice (see^13^ for a review). These previous studies have identified increases in spine turnover; in basal levels of corticosterone, glutamate, and in microglia motility^9,12,14,15^; meanwhile little is known about the acute effects of stress/corticosterone in this region.

Somatosensory cortex synaptic processing is affected by stress^9,14^. As local Field Potentials (LFPs) are thought to reflect the sum of synaptic activity^16^, it would be reasonable to expect stress induced changes to appear in S1 Local Field Potentials. However, a recent study found no differences in power spectral densities (PSDs) of LFPs recorded from S1 when chronically stressed and control animals were compared^11^. Here, we asked if the effects of stress could result in changes, not in the power of PSDs in the classical frequency bands (delta, theta, alpha, beta, and gamma), but instead in the dynamics resulting from the combination of specific ratios (Ratio1: 0.5–20 Hz/0.5–45 Hz; Ratio2: 0.5–4.5 Hz/0.5–9.0 Hz) organized in the form of state maps^17^. Analysis of changes in these ratios has previously allowed establishing parallels between LFP activity and tactile processing in awake behaving animals^18^, as well as the identification of general awake, sleep^17,19,20^, isoflurane anesthetized states^21^; or narcolepsy^22^. These parallels have been found for large networks encompassing multiple regions, as well as for single regions such as S1, thalamus, hippocampus, and caudate putamen^17,19,21^.

A relevant part of the neural response to stress seems to be associated with increased levels of glutamate, which are known to be neurotoxic^23^. As a result, translational activators of the excitatory amino acid transporters are now being used to reduce glutamate induced neurotoxicity in multiple animal models of disease^24–29^. For example, previous studies have demonstrated that ceftriaxone (a 3^rd^ generation cephalosporin that crosses the blood brain barrier) was able to improve the outcome of traumatic brain injury in rodents^25,26^. This suggested that ceftriaxone could potentially be used to treat or prevent the effects of stress responses in the brain, and more specifically in the primary somatosensory cortex.

Thus, in the present exploratory study we have tested two different hypotheses. The first hypothesis was that acute ceftriaxone administration was sufficient to modulate Local Field Potentials in the somatosensory cortex of anesthetized mice. The second hypothesis was that acute ceftriaxone administration would counter the effects of corticosterone induced modulations of S1 LFP activity. To test our hypotheses, we have recorded LFPs from the primary somatosensory cortex of four different groups of anesthetized mice injected with saline, corticosterone, ceftriaxone, and corticosterone with ceftriaxone.

## Results

**Figure 1:**
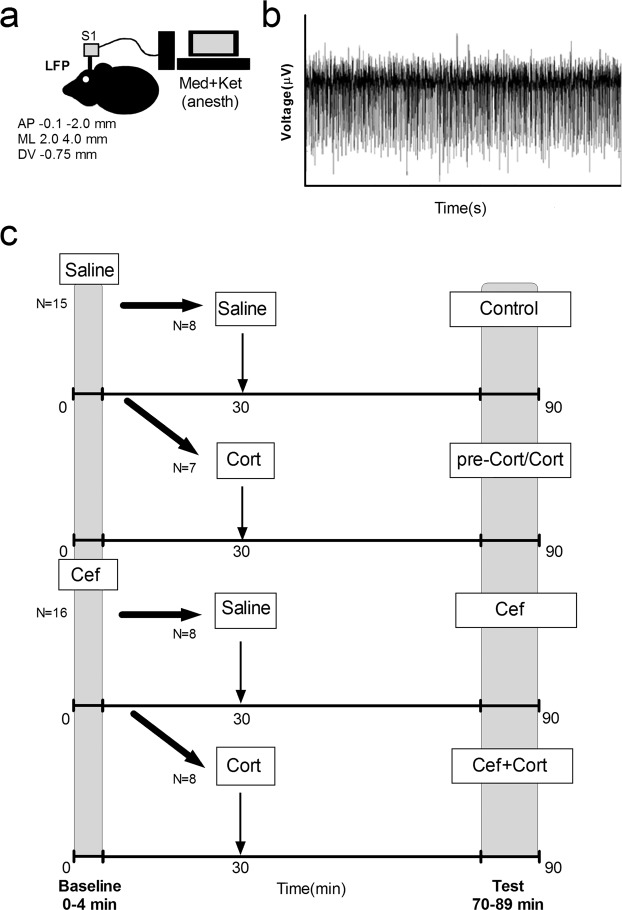
Experimental design. (**a**) Local Field Potentials (LFPs) were recorded from the primary somatosensory cortex of anesthetized mice. (**b**) Example of raw LFP trace. (**c**) Experimental design. An initial period (t = 0–4 minutes) and a testing period were defined (t = 70–89 min). Four different groups were studied: Control (injected twice with saline t = 0 and t = 30); Cort (injected with saline t = 0 and with corticosterone t = 30); Cef (injected with ceftriaxone t = 0 and with saline t = 30); and Cef + Cort (injected with ceftriaxone t = 0 and corticosterone t = 30).

A total of 32 mice were tested, from which 31 mice were used in the experiments reported here (n = 7–8 per group, 1 male mouse was excluded due to noise in recordings). Male and female (n = 3–4 male and n = 4 female, per group; in each mouse signals from two probes were analyzed) C57/Bl6 mice from Charles River laboratories with approximately 8–10 weeks old were used. We have analyzed four groups: Control, N = 8 mice (16 samples); Cef, N = 8 mice (16 samples); Cort, N = 7 mice (14 samples); and Cef + Cort, N = 8 mice (16 samples; also see Fig. 1a–c).

**Figure 2:**
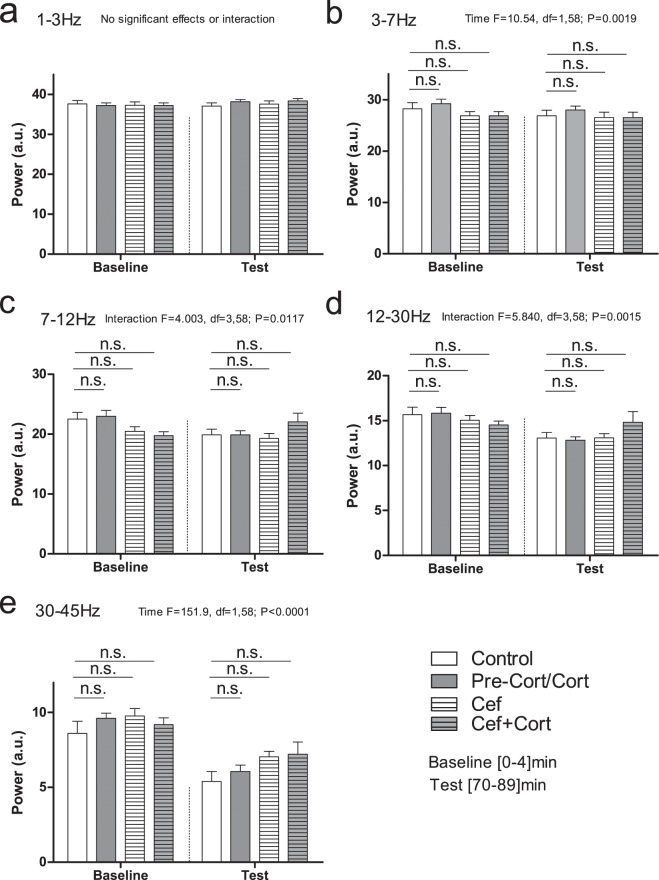
Power spectral densities for the main frequency bands. Panels a–e show the effects of ceftriaxone and corticosterone in the power of different frequency bands. No differences between groups were found for baseline or test periods, even though significant effects for Time or Interaction were found for particular frequency bands. Error bars indicate Mean ± SEM, n.s. indicates non-significant.

We started by asking if LFP power was similar between groups during the baseline and testing periods (see Fig. 2a–e). Comparison of power revealed significant effects of Time or Interaction (Time × Drug) in several frequency bands, however no significant differences were found when the different groups were compared with *post hoc* analysis. In the delta band frequency no significant effects were found (Time: F = 2.024; df = 1,58; P = 0.1601; n.s.; Fig. 2a). A significant effect of Time was found in theta frequency band (F = 10.54; df = 1,58; P = 0.0019; Fig. 2b). Significant Interactions (Time × Drug) were found in alpha (F = 11.01; df = 3,58; P < 0.0001; Fig. 2c), and in beta band frequency bands (F = 5.840; df = 3,58; P = 0.0015; Fig. 2d). Lastly, a significant effect of Time was found in gamma band (F = 151.9; df = 1,58; P < 0.0001; Fig. 2e). These results indicated that power changed for specific frequencies throughout the session, but no specific differences between groups could be identified during the baseline or test periods. Additionally, based on the variations between groups observed in Fig. 2 panel e, we pooled saline injected groups (i.e. Control and pre-Cort/Cort) and ceftriaxone injected groups (i.e. Cef and Cef + Cort) and compared power in the gamma frequency band. A significant Interaction was found (F = 4.808, df = 1,60, P = 0.0322, *post hoc* comparison for baseline period, P > 0.05; *post hoc* for test period P < 0.05; also see Supplementary Fig. S1).

We then asked if ceftriaxone and corticosterone could be changing broad states defined by ratios of LFP frequency bands. For this, we based our analysis on the previous study of Gervasoni and colleagues^17^, where two different ratios of frequencies were calculated (Ratio 1: 0.5–20 Hz/0.5–45 Hz; and Ratio 2 (0.5–4.5 Hz/0.5–9 Hz) and used to define broad state maps. The first ratio (Ratio 1) is the ratio of very low-middle frequencies (0.5–20 Hz) over very low-high frequencies (0.5–45.0 Hz). The second ratio (Ratio 2) is the ratio of very low frequencies (0.5–4.5 Hz) over low frequencies (0.5–9.0 Hz; Ratio2). As detailed in the methods section, we have calculated the average power for each of these ratios for the initial and the testing periods. The resulting values were then used to define the state maps.

**Figure 3:**
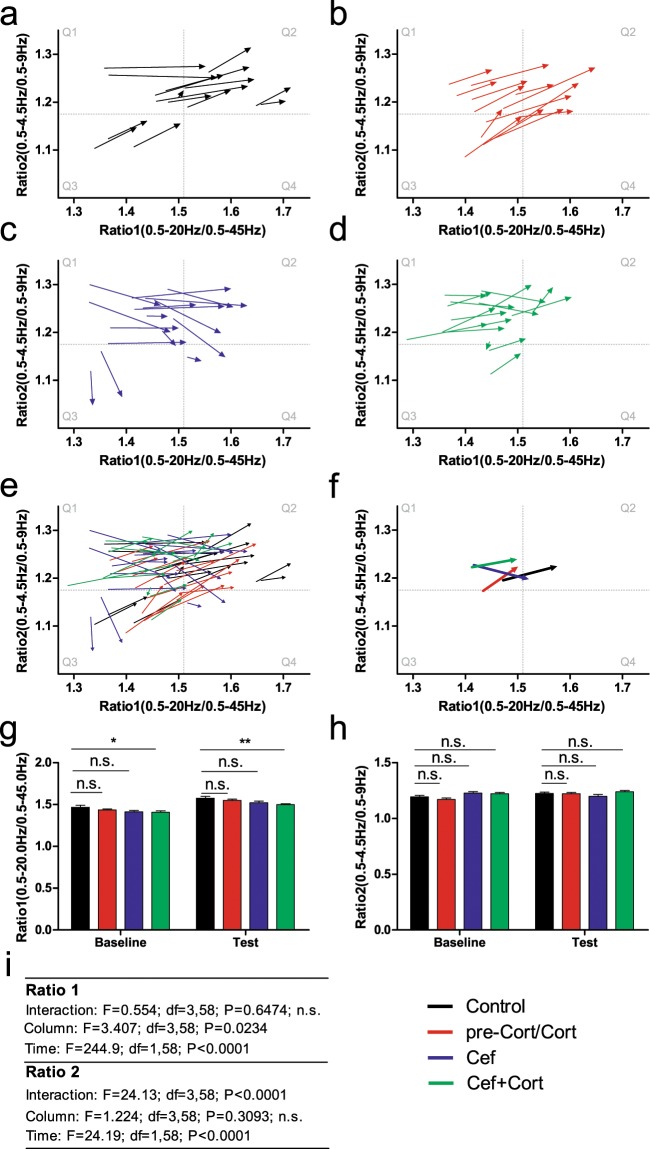
State maps defined by ratios of multiple frequency bands. State maps were defined according to the power for two different ratios: Ratio 1 (0.5–20/0.4–45 Hz) and Ratio 2 (0.5–4–5/0.5–9.0 Hz). Using these two different ratios, state maps with four different quadrants were calculated (Q1: top left; Q2: top right; Q3: Bottom left; Q4: bottom right). Each sample is represented with a vector (arrow). The vector is composed by two points. The first corresponds to the baseline values of each ratio for each sample. The second point (i.e. the arrow head) corresponds to the test period values. (**a–d**) Control, pre-Cort/Cort, Cef and Cort + Cef groups are depicted. The majority of the vectors point towards the right and up (in the direction of Q3). Meanwhile, vectors from the Cef group (blue) show an overall trend towards the right and down (also see f). (**e**) Vectors from all groups are presented simultaneously. (**f**) An average vector for each group is presented. As suggested by panels a-d, all groups with the exception of Cef presented an orientation towards Q3, while Cef presented an orientation towards Q4. (**g**) Comparison of baseline and test values for Ratio 1. The Control and Cef + Cort groups differed both in the baseline and the test periods. (**h**) Comparison of baseline and test values for Ratio 2, no significant differences between groups were found. (**i**) Detailed statistics for Ratio 1 and Ratio2. The presence of a significant effect for “Group” with significant *post hoc* analysis for baseline and test periods indicates that the Cef + Cort group cannot be compared using state maps. Error bars indicate Mean ± SEM, n.s. indicates non-significant. * and ** indicate P < 0.05 and P < 0.01, respectively.

The analysis of state maps for Ratio 1 and Ratio2 is presented in Fig. 3a–i. Left side quadrants (Q1 and Q3) reflect low values of Ratio1 (0.5–20 Hz/0.5–45 Hz) while right side quadrants reflect high values for Ratio 1 (Q2 and Q4). Top quadrants (Q1 and Q2) reflect high values for Ratio 2 (0.5–4.5 Hz/0.5–9 Hz) while bottom quadrants reflect low values for Ratio 2. Lastly, Q3 generally reflects low values in both Ratio1 and Ratio2, while Q2 will be associated with higher values of both Ratio 1 and Ratio 2. The data is presented as vectors starting at the coordinates of Ratio1 and Ratio2 during the baseline period and ending (arrow head) at the coordinates of Ratio1 and Ratio2 during the test period. Each group is represented in a different panel (Control: panel a; preCort/Cort: panel b; Cef: panel c; and Cef + Cort: panel d). Panel e depicts the vectors from all four groups studied and panel f shows the average of all vectors for each group. Panels g–i show the statistics related to Ratio1 and Ratio2 in each group.

Overall, most vectors from Control, pre-Cort/Cort and Cef + Cort groups presented a displacement towards the right and up. The exception to this was the Cef group, where vectors presented a displacement towards the right, but often also downwards.

In Fig. 3 panel g, the comparison of Ratio 1 for the different groups and phases of the sessions is presented. An overall effect for Groups was found (F = 3.406; df = 3, 58; P = 0.0234) which was significant only for the Cort + Cef injected group (*post hoc* Bonferroni, P < 0.05 for Cort + Cef in baseline and P < 0.01 for test period). This indicated that Control, pre-Cort, and Cef groups had similar baseline and test values for Ratio 1, but that the Cef + Cort group differed from Control group even during the baseline period. Meanwhile, a significant Interaction (Time × Drug) was found for Ratio 2 (F = 24.13; df = 3,58; P < 0.001), but no significant differences were found in *post hoc* analysis during the baseline or test periods (Fig. 3, panels h–i).

In short, the analysis of state maps indicated that: 1) for ratio1, the Cef + Cort group could not be directly compared to the Control group, because a significant difference was already present during the baseline period, and 2) the overall values of each ratio were similar between groups injected solely with ceftriaxone or corticosterone. Therefore, the effects of each individual drug could not be clearly dissected solely using the coordinates of the Ratio vectors in the state map.

**Figure 4:**
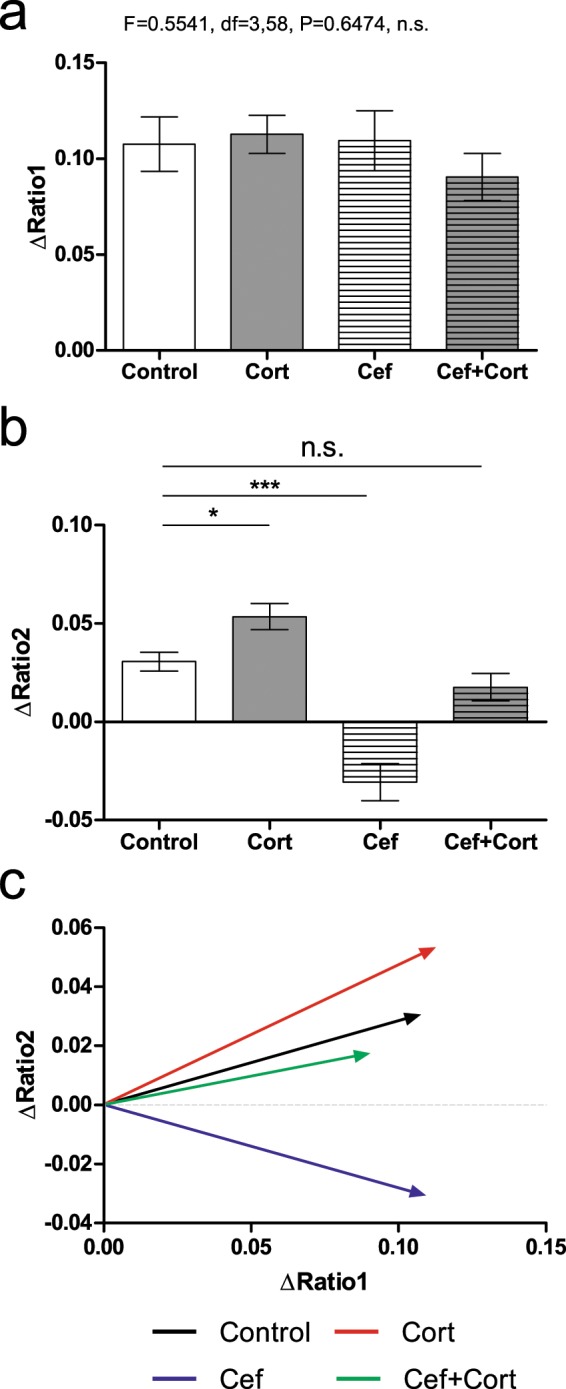
Ceftriaxone and Corticosterone induce opposite displacements in S1 state maps. (**a**) No significant differences were found between groups in ΔRatio1. (**b**) A significant increase was found in ΔRatio2 corticosterone injection (Cort), a significant decrease was found after ceftriaxone injection (Cef), and no significant difference was present when both ceftriaxone and corticosterone were injected (Cef + Cort). (**c**) Average displacement vectors representing the changes in ΔRatio1 and ΔRatio2 for each group. The Cef group presented a clear decrease in ΔRatio2. Error bars indicate Mean ± SEM, n.s. indicates non-significant. *indicates P < 0.05; ***indicates P < 0.001.

We then quantified the amount of displacement in each axis (i.e. the length of change in X and Y coordinates for each vector) throughout the session (Fig. 4a–c, also compare to Fig. 3, panel f). This is a relative measure of displacement (i.e. compares de actual distance regardless of where it started), and therefore is less sensitive to differences between groups that could be present in the initial period. To achieve this, we have analyzed three different measures of displacement: displacement in X axis (i.e. orientation and changes occurring in Ratio 1), displacement in Y axis (i.e. orientation and changes occurring in Ratio 2), and the Euclidean distance (absolute displacement in both axes simultaneously without regard for the specific orientation).

Displacement was calculated as the difference between the testing period coordinates for a particular axis, minus the initial period coordinate for the same axis (see methods for details). These values were termed as ΔRatio 1 and ΔRatio 2 respectively and they indicate how much the values of Ratio 1 (ΔRatio1: Ratio1_testing_ - Ratio1_initial_) or Ratio 2 frequencies (ΔRatio2: Ratio2_testing_ – Ratio2_initial_) have changed during a particular period. In Fig. 4a,b the values for ΔRatio1 and ΔRatio2 are depicted. Analysis of ΔRatio 1 revealed no differences between groups (F = 0.5541, df = 3,58; P = 0.6474). Meanwhile, analysis of ΔRatio 2 of lower frequency bands, revealed significant differences between multiple groups (F = 24.13, df = 3,58; P < 0.0001; *post hoc* Newman-Keuls: Control vs Cort: P < 0.05; Control vs Cef: P < 0.05; Control vs Cef + Cort: P > 0.05; n.s.) (see Fig. 4b,c). More specifically, corticosterone injection was associated with an increase in ΔRatio 2 when compared to Control group; while the Cef group was associated with the opposite effect and presented a decrease in ΔRatio 2. Most importantly, after both drugs were injected, no significant differences were found between the Cort + Cef and the Control groups. This analysis revealed that ceftriaxone and corticosterone had opposite effects in ΔRatio 2 and that these effects cancelled each other when both drugs were administered.

We then compared the overall displacement occurring in both axes simultaneously (i.e. the Euclidean distance). This measure accounted only for the actual distance of the displacement in both X (Ratio 1) and Y (Ratio 2) axes, but did not account for the specific direction of this displacement (e.g. increase or decrease in ΔRatio 1, ΔRatio 2 or both). No differences were found between groups (F = 1.082; df = 1,3; P = 0.3639; n.s.) indicating an overall similar amount of displacement among all groups.

Despite the small number of subjects in each group, we have also analyzed state map displacements in each sex. As presented in Supplementary Fig. S1, Male and female mice injected with ceftriaxone were differentially affected in ΔRatio 1 which is calculated using all frequencies between 0.5 and 45 Hz. Ceftriaxone increased ΔRatio 1 of Female Cef and decreased Male Cef (F = 3.885, df = 7,61; P = 0.0017; Bonferroni *post hoc*; Male Cef versus Female Cef: P < 0.05; all other comparisons n. s.). This was not the case for ΔRatio 2 were no gender differences were found (F = 9.818, df = 7,61, P < 0.0001; Bonferroni *post hoc*; all comparisons n.s.). As ΔRatio 2 was calculated solely using the lower frequencies between 0.5 and 9 Hz it is independent of ΔRatio 1, meaning that gender differences only affected ΔRatio 1.

## Discussion

Herein we describe the effects of administration of corticosterone and ceftriaxone, on local field potentials recorded from the primary somatosensory cortex of anesthetized mice. Analysis of broad state maps composed of ratios of multiple frequency bands (Ratio1: 0.5–20 Hz/0.5–45 Hz; Ratio 2: 0.5–4.5 Hz/0.5–9 Hz) demonstrated opposite dynamics for corticosterone and for ceftriaxone. More specifically an increase in the displacement of Ratio 2 (0.5–4.5 Hz/0.5–9 Hz) was found after corticosterone administration, while a decrease was found after ceftriaxone administration. These opposite effects cancelled each other when both drugs were administered, with the displacement values of ΔRatio 2 becoming similar between the Cef + Cort group and the Control group.

Our first hypothesis was that acute ceftriaxone administration was sufficient to modulate LFPs in the somatosensory cortex of anesthetized mice. This hypothesis was confirmed, since ceftriaxone injection induced changes in LFP in state map displacements. Ceftriaxone has been previously demonstrated to enhance glial glutamate transporter (GLT-1), therefore having a neuroprotective role in traumatic brain injury^26^, ischemia^27^, cocaine dependence^28^, neuropathic pain^30^, and multiple sclerosis^30^.

However, this mechanism involves glial modulation and, to our best knowledge, has only been described to occur after repeated administration of ceftriaxone (see^29^ for a review). Therefore, it was not clear *a priori* that ceftriaxone should induce changes in LFP recordings in such a short amount of time (90 minutes), and much less clear, that these effects should largely be opposite to those of corticosterone. In upcoming studies it will be important to determine if the opposing effects for ceftriaxone and corticosterone reported here are related to the mechanism previously described for GLT-1 and corticosterone dendritic spine remodeling^9,31^, or if they constitute a different one.

Our second hypothesis, that acute ceftriaxone administration would counter the effects of corticosterone induced modulations of S1 LFP activity, was also supported, since ceftriaxone prevented the effects of corticosterone in state map dynamics. Previous studies, related to the development of S1, have reported multiple long-term changes in neural activity following stressful events. These changes included increases in spine turnover; in basal levels of corticosterone, glutamate, and in microglia motility after stress^9,12–15^. Despite all of these changes, a previous study with chronic corticosterone administration, found (as we have here), no effects of corticosterone in the LFP power when specific frequency bands were analyzed^11^. Our present results indicate that corticosterone induced changes in S1 state map dynamics rather than in specific frequency bands.

Corticosterone injection was associated with an overall effect opposite to that of ceftriaxone, namely an increase in ΔRatio2 (mostly related to delta and theta frequency bands). Therefore, corticosterone injection induced changes that suggest a transition towards a state characterized by lower frequencies. Lower frequency oscillations in the somatosensory cortex have been previously described to occur in synchronized states. Neuromodulators such as noradrenalin (through the Anterior Nucleus Basalis of the Locus Coeruleus), and GABA (due to the effect of the Reticular Thalamic Nucleus) have been associated with an overall shift towards lower frequency oscillations in the somatosensory cortex^32,33^. It thus remains to be explained if the present observations of opposite effects for ceftriaxone and corticosterone are related to neural substrates previously described for high and low oscillation states^34^, or glial glutamate transporter^31^ and spine remodeling^9^.

All groups presented a decrease in Ratio 1 (composed mostly by beta and gamma frequency bands) throughout the course of the session, suggesting either an increase in gamma frequency band power (i.e. an increase in the denominator of Ratio2) or a decrease in beta band power (i.e. a decrease in the numerator). Comparison of gamma band power for data pooled from ceftriaxone injected groups (i.e. Cef and Cef + Cort) or saline injected groups (i.e. Control and pre-Cort/Cort) suggested that Ceftriaxone may be associated with increased power in gamma frequency band. Gamma rhythm controls sensory responses in rodents^17,35–37^ and humans^38,39^, and is known to involve thalamo-cortical as well as cortico-cortical connections with glutamatergic and cholinergic components^40,41^. Somatosensory cortical states characterized by higher LFP frequencies have been previously reported to occur in rodents after thalamocortical, cortico-cortical and brain stem activity^34^. Namely, stimulation of the primary motor cortex, basal forebrain (through cholinergic activation) or thalamic relay nuclei (through glutamatergic activation) have all led to desynchronized states, which are typically associated with increased power in higher frequencies^32,34,40–47^. Additional studies will be required to precisely determine if the effects of ceftriaxone occur in gamma or in beta bands.

In the original study of Gervasoni and colleagues^17^, state maps were used to describe the neural substrate of particular behaviors/general states such as whisking, grooming or Rapid Eye Movement sleep. In the present study, mice were anesthetized, for what our state maps differed from the ones described by the original authors. However, as the analysis of the displacement ratios allowed dissecting the different effects of each drug on LFP activity, this indicates that this modified version constitutes a relevant technique for future studies on LFP modulations of cortical states. We propose that this analysis may be particularly suitable for real-time processing of LFP data (such as in brain-machine interface applications), where computational power is often a critical variable.

The results reported here support the notion that a potential neuroprotective role for ceftriaxone in stress should be further explored, however as we have tested anesthetized mice without actual tactile stimulation, the neuronal dynamics described here may differ from the ones observed in awake rodents^17^. Also, models of acute and chronic stress display multiple neurophysiological, neurochemical and connectome profiles, with some of them being adaptive and other maladaptive^2,48^. It will be important for future studies to determine if the neuroprotective effects of ceftriaxone reported here are also present in other models of acute and chronic stress.

Generalization of the present findings to other contexts should be made with particular care due to the following technical limitations. First, the results from the present study, were collected in a relatively small mixed sample (total of 14–16 samples per group, with 7–8 mice per group). Second, we have not recorded data from a “true baseline” period, where no manipulation was performed. This means that we cannot ascertain that groups were not different when the experimented started. Third, we have tested adolescent mice. This prevents generalization of our findings to other ages. Fourth, we have found different effects for ceftriaxone and corticosterone in two ratios of frequency bands. This is a very specific measure of brain activity which is difficult to compare with other, more common measures (e.g. increases or decreases in a particular frequency band). Fifth, male and female mice were used here. Even though no clear gender differences were found for ΔRatio 2 (i.e. the main finding of this study), one cannot exclude this possibility if a larger sample is analyzed. This is of particular importance since a previous report has found differences in LFPs recorded from the somatosensory cortex of male or female mice^49^. Further studies specifically comparing larger samples of male or female mice in adulthood will be required to consolidate the findings of the present exploratory study. Finally, the analysis of ratios of frequency bands in previous studies was directly associated with simultaneous analysis of other measures such as sleep characteristics or exploratory behavior^17,19,20,22^. Here however, we have analyzed the dynamics of LFP activity, but no specific behavior or any other physiological measure were evaluated. It is therefore not known if the LFPs maps’ presented here actually represent cortical state maps as defined in previous studies.

One of us has previously proposed a model to explain the changes occurring in the brain during the transition from acute to chronic stress, the stress neuromatrix^2^. According to such model, critical nodes and/or chemical markers may be involved in the transition between adaptive and maladaptive stress responses^2^. The present study describes a stress hormone modulation that can be partially prevented pharmacologically, therefore it establishes a starting point for future studies unraveling the role of the trigeminal system in acute and chronic stress.

On a broader perspective, our findings suggest that the use of pharmacological manipulation of glial cells^50^ may be useful in the treatment of other functions known to be affected by stress. For example, it will be important to test the effects of ceftriaxone in motor^51^ and cognitive functions^52,53^ of stressed animals.

## Conclusion

We have described here the effects of corticosterone and ceftriaxone in local field potentials recorded from S1. The present results support the notion that ceftriaxone may be a suitable candidate to reduce, treat or prevent the effects of corticosterone.

## Methods

The animal facilities and the personnel involved in the experiments are certified by the DGAV (Direção Geral de Alimentação e Veterinária – DGAV 023432). The present experiments were performed in accordance with the European Union’s regulations (European Union Directive 2010/63/EU) as well as FELASA’s guidelines for housing, manipulation, and experimentation^54^. The procedures and protocols were approved by the Ethics Committee of the Life and Health Sciences Research Institute (ICVS).

In Fig. 1(a–c) we present the experimental design used here. Sessions lasted for 90 minutes. Each mouse was injected twice during a recording session. The first injection was IP and was delivered at T = 0 (immediately before starting the recording), the second injection was SC (in the dorsum) and was delivered at T = 30. Recordings were then interrupted at 30 minutes to perform the second injection. Four different groups were formed: Control (N = 8), Corticosterone (N = 7; 25 mg/kg, subcutaneously (SC), dissolved in sesame oil; Sigma Aldrich, St. Louis, MO, USA), Ceftriaxone (N = 8; 200 mg/kg, intraperitoneally (IP) in water, Merck, NJ, USA), and Corticosterone + Ceftriaxone (N = 8). As depicted in Fig. 1c, mice in the Control group (Control) were injected with saline a time T = 0 and at time T = 30 minutes. Mice in the Corticosterone group (Cort) were injected with saline at time T = 0 and with corticosterone at time T = 30. Mice in the Ceftriaxone group (Cef) were injected with ceftriaxone at time T = 0, and with saline at time T = 30. Lastly, mice in the Ceftriaxone + Corticosterone (Cef + Cort) group were injected with ceftriaxone at time T = 0 and with corticosterone at time T = 30. These specific times were used to facilitate interaction between the two drugs during the testing period.

We have included male and female mice to ensure both genders were represented in the final conclusions of the present study. As a previous study^9^ has demonstrated that glucocorticoids are critical for spine formation and elimination in adolescent mice, we have opted to test mice at the end of adolescence (8–10 weeks).

### Surgery

Mice were anesthetized with a mixture of medetomidine (1 mg/kg, IP) and ketamine (75 mg/kg, IP). After mice were injected with the anesthesia mixture, they were kept in a dark environment until no pain reflexes were present. Ointment was used to protect the eyes and mice were then placed in the stereotaxic frame. After proper disinfection, skin and underlying tissues were sectioned and separated. A window was then marked around the coordinates −0.1;−2.0 mm AP; 2.0–4.0 mm ML; and −0.750 mm DV^55^ and the bone was drilled. After bone removal, the dura mater was carefully removed and the somatosensory cortex (S1) exposed. An additional ground screw was placed in the occipital bone. Lastly, a multielectrode array with two probes (separated by 250 micrometers; aligned in the anterior to posterior direction) was lowered at the center of the window. We have opted here to analyze LFP data from wires at depths corresponding approximately to layer V (750 µm). It should be noted that we were not able to perform appropriate histological verification due to the reduced size of our probe (~25 µm).

### Recordings

An Open Ephys system with 64 channels was used here. Signals were initially collected using a Cambridge Neurotech probe (ASSY 77H-H2) (32 wires 25 micrometers apart of each other in the vertical direction per probe, in two different probes separated by 250 micrometers). The signal was digitized at 30000 Hz and saved for later analysis. Data was then parsed and low pass filtered at 400 Hz and analyzed in Neuroexplorer 5 (Nex technologies, USA), GraphPad Prism 5 (GraphPad Software, Inc.; USA), and Matlab R2013b (Mathworks, USA).

The initial period corresponded to the first 4 minutes of the session, the testing period corresponded to the last 19 minutes of the session. These particular intervals were related to the number of samples analyzed (i.e. choosing a round number of samples). Power spectra were calculated using Neuroexplorer V5 with LogPSD normalization and Gauss smoothing of 3 bins with a 50% overlap. These values were then exported and statistical analysis performed in GraphPad and Matlab R2013b. Comparison of differences between groups during the baseline and test period (i.e. between Control, Cort, Cef and Cef + Cort groups) was performed with a Two Way repeated measures (Drug × Time) ANOVA followed by Bonferroni *post hoc* analysis.

### State maps

Analysis of state maps was based on a technique described in a previous study^17^. This technique uses two different ratios (Ratio 1: 0.5–20 Hz/0.5–45 Hz; and Ratio 2 (0.5–4.5 Hz/0.5–9 Hz) to describe broad states. We will refer to each interval of frequencies as follows: 0.5–4.5 as low-frequencies; 0.5–9.0 as middle-low frequencies; 0.5–20 as middle-high frequencies; 0.5–45 as high-frequencies. It should be highlighted that despite this terminology, each group contains all the lower frequencies (for example, middle-high frequencies include low and middle-low frequencies). The terms “increased” and “decreased” will be used throughout the manuscript to indicate relative measures comparing the changes in a particular variable of a group (e.g. Ratio 1) between the baseline and the test periods.

Here we have adapted the original technique (which was based on Principal Component Analysis) and used a computationally less intensive technique. For this we have used the average power for each group of frequencies. To calculate the extremes (highest and lowest values) and midpoint of the quadrants (Q1, Q2, Q3, and Q4) we have considered data from all groups.

### State map displacements

State map displacements were calculated using the first derivative of state maps. The goal of this analysis was to quantify changes that occurred in state maps throughout a session, without requiring groups to have similar baseline values. Thus, this analysis described the amount of change that occurred within each group, rather than comparing the actual values of each ratio. We will start by describing the calculations performed for ΔRatio 1. ΔRatio 1 values were calculated for each specific probe as the difference between each value calculated for Ratio 1 at the testing period and the value calculated for Ratio 1 during the initial 4 minutes (ΔRatio1: Ratio1_testing_ - Ratio1_initial_). These values were then studied using an ANOVA (or Kruskal-Wallis test, when no homogeneity of variance) followed by *post hoc* comparisons with Newman-Keuls or Dunn’s test (when no homogeneity of variance was found). The same calculations were then applied to Ratio2 (ΔRatio2: Ratio2_testing_ - Ratio2_initial_). We have used here data from two probes in each mouse since neural activity in state map displacements was not correlated for probes recorded in the same hemisphere in control mice.

To study the overall amount of displacement in both ratios simultaneously, regardless of the specific direction (positive or negative in each axis), Euclidean distances were compared. Euclidean distances were calculated as the square root of the sum of the vector length composed by values of ΔRatio1 (first coordinate) and ΔRatio2 (the second coordinate). These values were then analyzed using a One Way ANOVA (or Kruskal-Wallis test, when no homogeneity of variance was present) to compare the effect of each Drug.

## Supplementary information

Supplementary Figures S1 and S2.

## Data Availability

The datasets generated during and/or analyzed during the current study are available from the corresponding author upon reasonable request.
