# Supplementary material for: Ceftriaxone modulates the acute corticosterone effects in local field potentials in the primary somatosensory cortex of anesthetized mice
Source: Sci Rep. 2019 Dec 30;9:20289. doi: 10.1038/s41598-019-56827-8 (PMC6937346; doi:10.1038/s41598-019-56827-8)
Supplement: Supplementary file 1 — Supplementary Figures S1 and S2. [file 41598_2019_56827_MOESM1_ESM.pdf]

Ceftriaxone modulates the acute corticosterone effects in local field potentials in the primary somatosensory cortex of anesthetized mice

Miguel Pais-Vieira, Carolina Kunicki, André Peres, Nuno Sousa

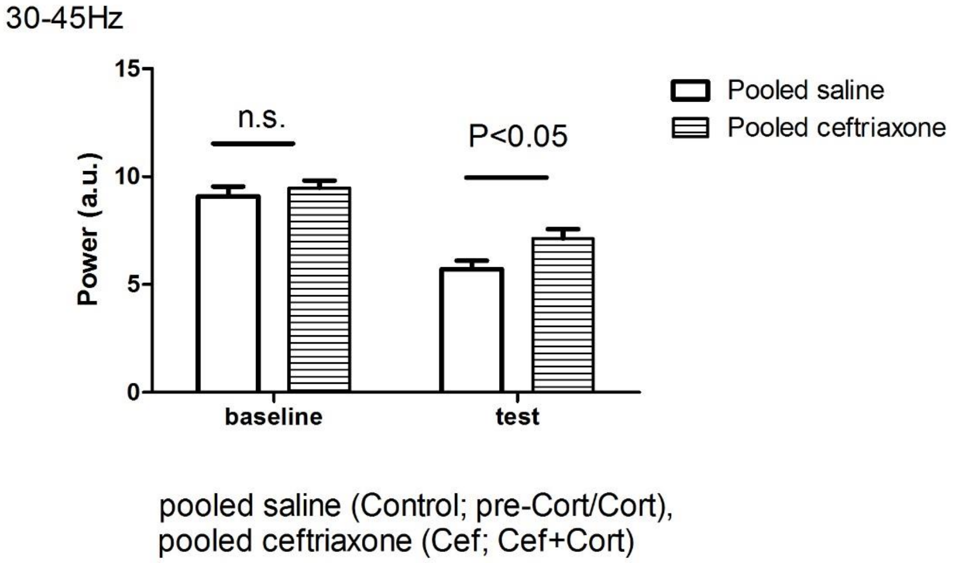

Supplementary Figure S1: Comparison of Ceftriaxone and Saline effects in pooled groups. Comparison of groups injected with saline (Control and pre-Cort/Cort) or injected with ceftriaxone (Cef and Cef+Cort) suggests that ceftriaxone may be increasing power in the gamma frequency band. Error bars indicate Mean±SEM, n.s. indicates non-significant.

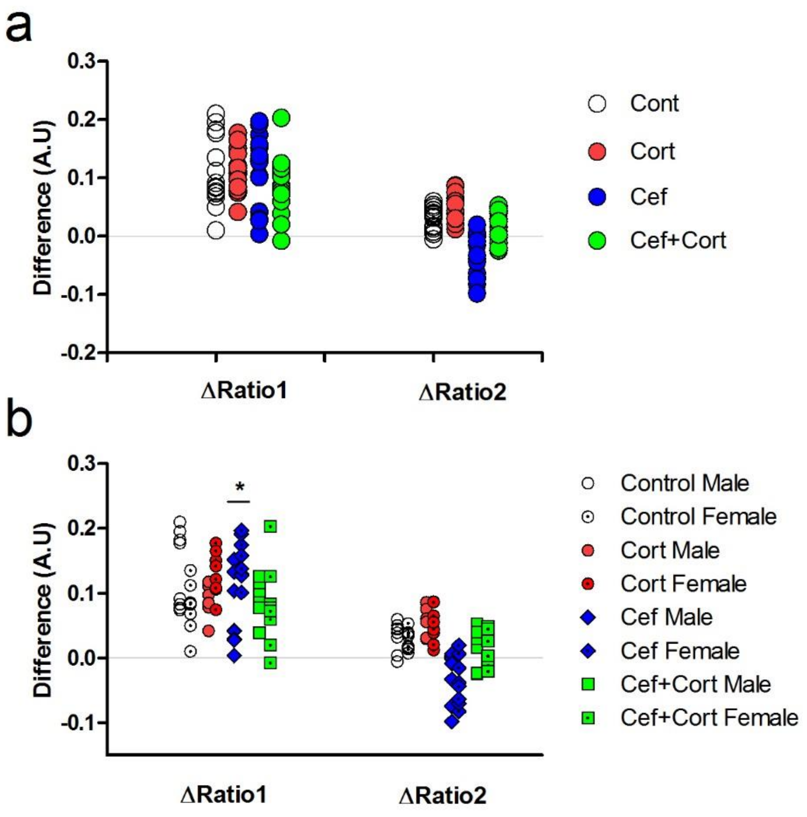

Supplementary Figure S2: Comparison of state map discplacements according to gender. a) ΔRatio 1 and ΔRatio 2 displacements. b) Gender comparison of ΔRatio displacements. A significant effect was found when male and female samples were compared, suggesting that ceftriaxone may have opposite effects in ΔRatio1. No significant differences were found in ΔRatio 2. * $P<0.05$for *post hoc* comparison.
